# 2285 Patient navigation training: Community-engaged workforce development

**DOI:** 10.1017/cts.2018.295

**Published:** 2018-11-21

**Authors:** Nirmal Ahuja, Joanne Sullivan, Eugene Lengerich

**Affiliations:** Clinical and Translational Science Institute, Penn State University

## Abstract

OBJECTIVES/SPECIFIC AIMS: The goal of this initiative was to address this cancer health disparity in the Appalachian counties and help participants develop, implement and evaluate evidence-based “PN” that effectively and positively impacts patient and outcomes of the HealthyWomen Program. Following were the objectives of this training program: (1) To understand the broad range of roles and responsibilities associated with “PN”, including care coordination and case management, in the Pennsylvania HealthyWoman Program in Pennsylvania and the National Breast and Cervical Cancer Early Detection Program. (2) To identify and assess local resources and expertise for evidence-based “PN” in the HealthyWoman Program. (3) To utilize “PN” in association with public education and targeted outreach initiatives in the HealthyWoman Program. (4) To implement strategies to manage and evaluate “PN” for the HealthyWoman Program. METHODS/STUDY POPULATION: The series of PN training was held at Pittsburgh, Camp-Hill, Wilkes-Barre and Philadelphia during June 2017. In total, 86 participants attended the training program at one of these 4 locations. Attendees represented organizations that provided breast, cervical and colorectal cancer outreach, screening and treatment. The participants of the training were solicited by regional program managers of the HealthyWoman Program of the PA Department of Health. The Harold Freeman model for patient navigation model was used to train the participants on the concepts of patient navigation. The training was built upon the Health Belief Model and Chronic Care Model, which defined the specific program constructs. The curriculum covered 2 important aspects, that is, clinical knowledge related to breast and cervical cancer along with aspects of patient navigation. Participants represented small, and large academic institutions/health care systems, cancer centers, federally qualified health centers, health departments as well as community-based screening programs and organizations. RESULTS/ANTICIPATED RESULTS: A total of 86 participants were trained; 78% had formal education and training in health-related field. In total, 62% of the participants had previous experience of patient navigation; 42% had training in social service related field and 50% had prior experience as community health worker. The demographic details reflected that majority of the participants (94%) were female. Most of the participants (30%) belonged to 50–64 years of age group followed by 30–39 years (23%) and 40–49 years (22%) of age group, respectively. As part of ethnic distribution, 70% of the participants were White Americans followed by Black/African Americans (17%). Furthermore, association of previous training in health and social service field with and without experience as a community health worker (n=84) and Navigator (n=86) was also analyzed. Among the participants, 44% had both community health worker experiences along with a prior training in social service related field whereas 42% of the respondents only had prior social service related training. This association of previous training in social service related field and prior community health worker experience was statistically significant with a *p* value of <0.05. Additionally, 81% of the participants who had previous experience in health-related training also possessed the prior experience as community health worker. Also, 81% of the participants who had previous training in health-related field also had a prior experience of patient navigation. In all, 38% of the participants who had a previous experience in social service field also had a prior experience of patient navigation. DISCUSSION/SIGNIFICANCE OF IMPACT: The training program established a pool of patient navigators which will contribute towards reducing the cancer health disparity in the Appalachian region of Pennsylvania. The participants reflected a wide diversity in the navigators’ backgrounds and differences across programs in their choices of patient navigators. It is important to consider this diversity when designing curricula materials and the methods of delivery in a patient navigation training program. As PN training programs are developed and implemented, further data is needed to guide practitioners and administrators in their efforts to include separate curriculum and materials for experienced and lay navigators. In addition, it is also important to assess the role and involvement of patient navigators in research and clinical trials. In total, 82% of the participants when asked agreed to be contacted for participation in research studies. Specific curriculum which includes research could be designed for further development of patient navigators. PN training and implementation knowledge is critical to the development of standards and best practices in this emergent area of cancer care.

